# Cervicogenic Angina and Dyspnea Secondary to Cervical Radiculopathy

**DOI:** 10.7759/cureus.37515

**Published:** 2023-04-13

**Authors:** Eric Chun-Pu Chu, Steve Yun, Kevin Hsu Kai Huang

**Affiliations:** 1 Chiropractic and Physiotherapy Centre, New York Medical Group, Hong Kong, HKG

**Keywords:** angina pectoris, cervical radiculopathy, cervicogenic dyspnea, cervicogenic angina, chiropractic

## Abstract

Cervicogenic angina and dyspnea are conditions characterized by chest discomfort and breathing difficulties that resemble angina pectoris and dyspnea of cardiac origin. However, this condition is caused by cervical spine pathology, cervical spondylosis, and radiculopathy. This case study reports a 66-year-old man who presented with cervicogenic angina and dyspnea due to cervical radiculopathy to a chiropractic clinic. The patient underwent a comprehensive diagnostic evaluation, including taking the patient’s history, a physical examination, and radiological investigations, which demonstrated cervical spine involvement consistent with a cervicogenic origin of the pain. The patient's angina-like symptoms and dyspnea improved significantly after chiropractic manipulation of the spine, soft tissue mobilization, and other manual therapies. Accurate diagnosis is essential to minimize unnecessary cardiac interventions and ensure proper therapy for underlying cervical spine problems. This case demonstrates the necessity of conservative management, such as chiropractic care, for patients presenting with cervicogenic angina and dyspnea, particularly when a diagnostic assessment reveals cervical spinal involvement.

## Introduction

Cervicogenic angina refers to chest pain and discomfort mimicking angina pectoris but is caused by cervical spine pathology, particularly cervical spondylosis and radiculopathy [1]. The pain is usually associated with neck pain and can be exacerbated by cervical movements, resulting in dyspnea, palpitations, blurred vision, dysphagia, and dizziness [2-10]. Cervicogenic dyspnea is a less commonly reported symptom of cervical spinal dysfunction, leading to breathing difficulties [11]. The exact mechanisms underlying cervicogenic angina and dyspnea are not fully understood; however, cervicogenic angina is most frequently linked to radicular discomfort in the lower cervical spine [12].

Accurate diagnosis of cervicogenic angina and dyspnea is essential for the appropriate management of the symptoms and underlying cervical spine disorder and the prevention of unnecessary cardiac interventions [1]. While dyspnea and acute chest pain are two of the most prevalent non-traumatic signs that elicit emergency room examinations, there is considerable overlap between these presenting symptoms. Each symptom necessitates a broad differential diagnosis list that must be quickly narrowed down using information from the patient’s history, clinical examination, laboratory results, and radiographic analysis [13].

Chiropractic care, including spinal manipulation, soft tissue mobilization, and other manual therapies, has been reported to provide relief for patients with cervicogenic angina and dyspnea [12, 14]. Moreover, conservative management of cervical radiculopathy, including chiropractic treatment, has been shown to improve pain, function, and quality of life in affected individuals [15]. This case report outlines the successful chiropractic management of a 66-year-old man who presented with cervicogenic angina and dyspnea secondary to cervical radiculopathy.

## Case presentation

A 66-year-old man with a 12-month history of worsening left temporal headache, dyspnea, chest discomfort, and neck pain and a three-month history of left ulnar neuropathy presented to a chiropractic clinic. He rated his pain symptoms as four out of 10 on the numeric rating scale and 78/100 using the World Health Organization Quality of Life (WHOQOL) scoring tool. The patient had not experienced any recent trauma or cardiovascular events, although he had a family history of diabetes and heart disease. His symptoms gradually worsened, and he experienced occasional palpitations but denied orthopnea or edema.

He was initially reviewed by his family doctor and underwent stress echography, electrocardiography, and cardiac enzyme examinations, which were unremarkable apart from hyperglycemia. He was diagnosed with ischemic heart disease and diabetes mellitus type 2 and was administered aspirin, atorvastatin, metoprolol, and oral hypoglycemics. However, his dyspnea, headache, and neck pain worsened significantly over the following three months, and he developed numbness and paresthesia in the lateral third and fourth digits of his left hand. The patient was unable to participate in previously enjoyed hobbies or recreational activities. A second opinion was obtained from an orthopedic surgeon, and cervical radiculopathy was confirmed based on his history, physical examinations, and radiological findings. He was on analgesia and physical therapy, which provided temporary relief. Owing to inadequate symptom control and the impact on his quality of life, he presented to us seeking chiropractic care for further management.

**Figure 1:**
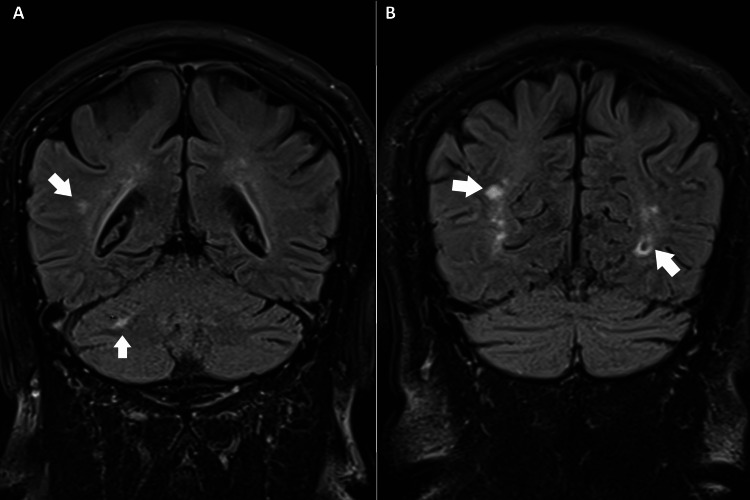
Magnetic resonance imaging (MRI) of the brain A and B) Multiple non-specific, tiny fluid-attenuated inversion recovery (FLAIR) hyperintense foci (white arrows) are observed in the subcortical white matter of the supratentorial brain.

**Figure 2:**
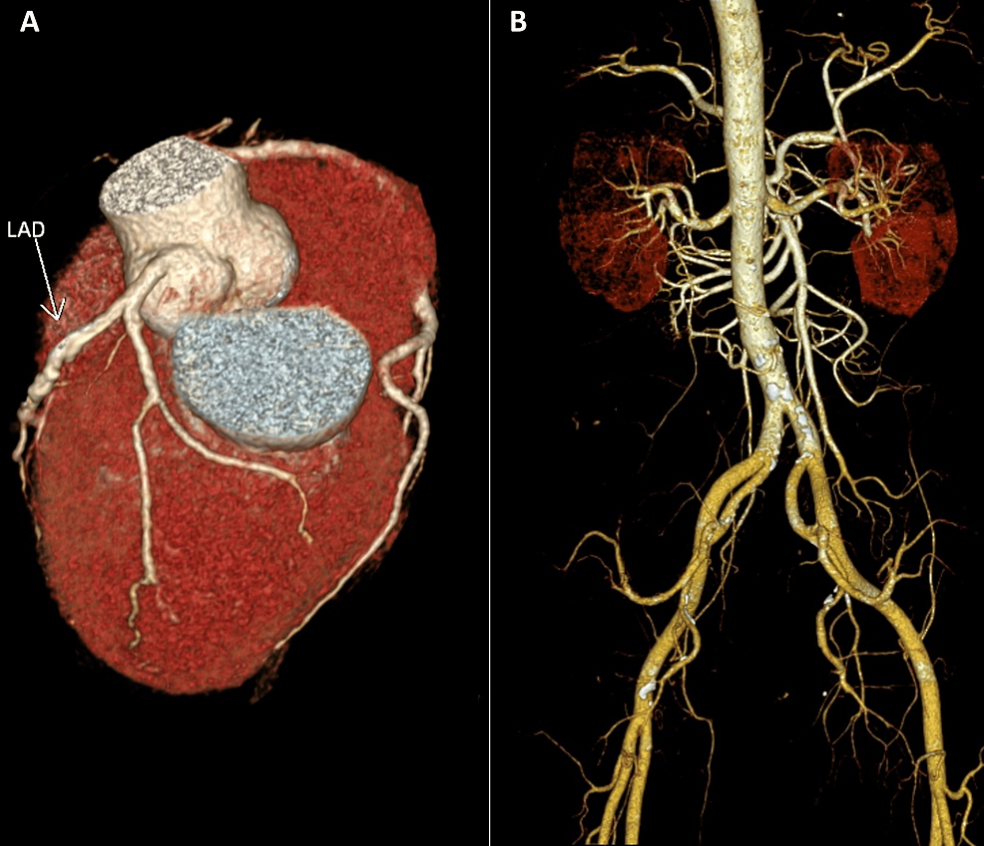
Coronary and abdominal computed tomography (CT) angiography A) Moderate stenosis is noted at the proximal left anterior descending artery (LAD) with calcified plaques (white arrow), at the medial LAD with mixed plaques, and mild stenosis at the distal LAD with calcified plaques. B) Atherosclerotic plaques are observed along the abdominal aorta and bilateral common iliac, bilateral internal iliac, and left common femoral arteries. Abdominal angiography is unremarkable

**Figure 3:**
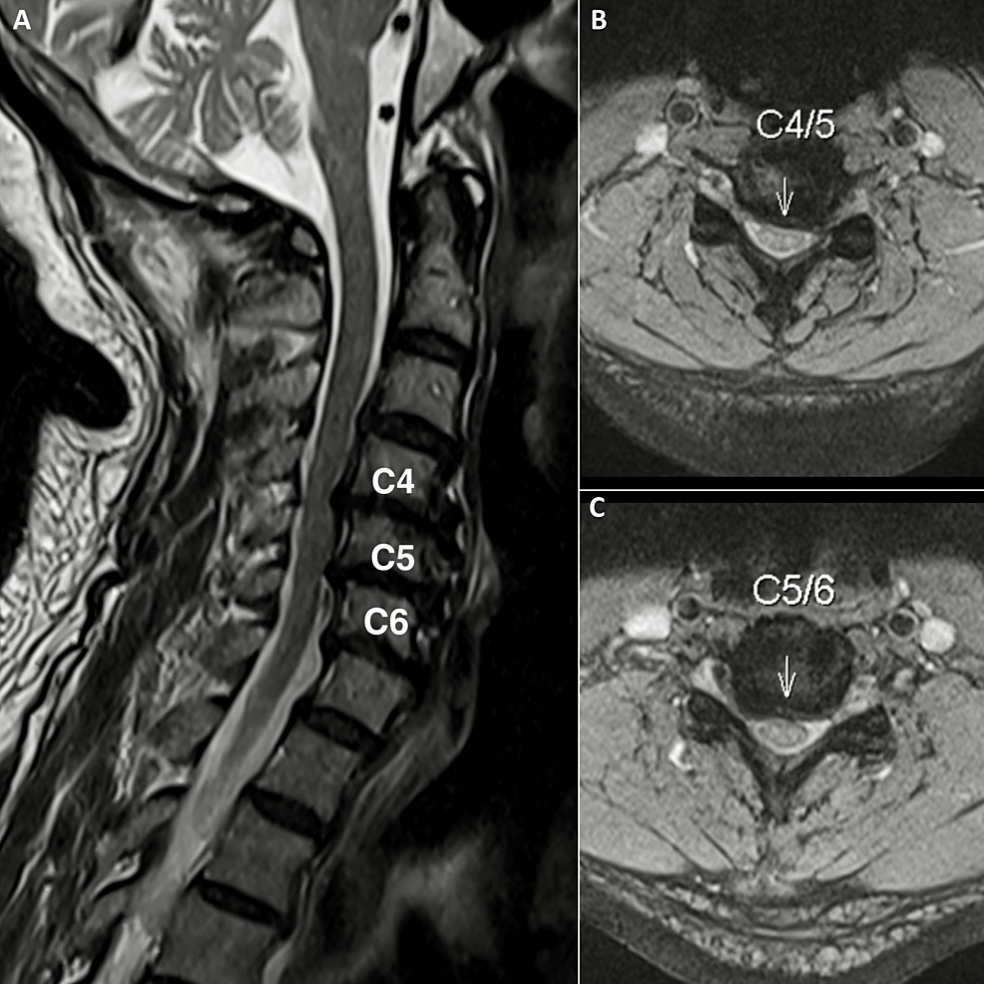
Magnetic resonance imaging of the cervical spine A) Cervical spondylosis with diffuse disc desiccation and decreased disc height at C4/5, C5/6, and C6/7 levels. B) Spinal stenosis at C4/5 and C5/6 levels with cord impingement but no abnormal cord signal.

Orthopedic examination of the cervical spine revealed hypertonicity of the bilateral trapezius, left sternocleidomastoid, pectoralis major, and left levator scapulae muscles, with a restricted range of motion limited to 20° of extension and 40° of bilateral rotation (normal: > 70° and > 90°, respectively). Spurling's test reproduced chest discomfort and paresthesia in the lateral third and fourth digits of the left hand. Neurological evaluation revealed 4/5 strength in the left upper extremity and diminishing sensation in the left C6 and C7 dermatomes. Chiropractors’ differential diagnoses included cervical artery dissection, cervical angina, tension and migraine headache, brain aneurysm, vertebral artery aneurysm, and other potentially life-threatening conditions that were excluded. Advanced imaging examinations were performed immediately. Brain magnetic resonance imaging (MRI) with magnetic resonance angiography (MRA) showed insignificant, nonspecific fluid-attenuated inversion recovery (FLAIR) hyperintensities in the subcortical white matter of the supratentorial brain (Figure 1). Coronary and abdominal computed tomography show moderate stenosis (Figure 2). However, the cervical MRI demonstrated multilevel cervical spondylosis with disc space narrowing and desiccation at C4/5, C5/6, and C6/7 and spinal stenosis with cord abutment at C4/5 and C5/6 without signal abnormalities (Figure 3). The clinical presentation and laboratory and imaging studies confirmed a diagnosis of cervical radiculopathy at the C6/7 level with concomitant cervicogenic angina and dyspnea.

As adverse events following chiropractic treatments are very rare [16], chiropractic spinal manipulation was integrated into the patient’s treatment plan to address cervical joint dysfunction and instrument-assisted soft tissue mobilization (IASTM) to relax the hypertonicity of muscles. Treatment was administered three times per week for the first month. He had a 70% improvement in symptoms within the first two weeks, with a decrease in the pain score from four to one. At one month, intermittent motorized traction was applied to help distract the intervertebral disc and release nerve root impingement. The treatment frequency was reduced to twice a week for two months. At the three-month re-evaluation, he reported complete recovery of cervical/chest pain, dyspnea, and paresthesia, with improved range of motion. Analgesia was discontinued, a self-care program was implemented, and the patient will be followed up on an as-need basis for chiropractic maintenance care. At the 12-month visit, his WHOQOL score improved to 100, and he remained asymptomatic without adverse effects.

## Discussion

Management of cervicogenic angina and dyspnea is a complex process, as their similarities with cardiac-related conditions lead to diagnostic challenges [2]. The patient, in this case, reported experiencing significant improvements in angina-like symptoms and dyspnea following chiropractic management, highlighting the potential benefits of conservative treatment for cervical radiculopathy [14]. The current literature supports the use of spinal manipulation and other manual therapies for the treatment of cervicogenic symptoms, including headaches, dizziness, and angina [15,17].

The first proposed mechanism for cervicogenic angina and dyspnea involved the compression of the cervical nerve roots [18]. Approximately 70-72.6% of cervicogenic angina cases are caused by compression of the cervical nerve root, particularly the C4-8 (prefixed brachial plexus) [12]. The brachial plexus, which supplies sensory and motor innervation to the pectoral girdle and upper extremities, originates from the cervical nerve and travels through the first rib and the axillae via the cervicoaxillary canal [19]. This complex neurological network is composed of the anterior branches of the C5-8 and T1 nerves, which are typical plexus components [20]. The plexus can be anatomically classified into prefix (C4-8) and postfix (C6-T2) varieties [12]. The prefixed brachial plexus affects the majority (26%-48%) of people, and the postfixed brachial plexus is only found in 4% of people [12]. The prefixed brachial plexus innervates the lateral and medial pectoral nerves to the pectoralis major, pectoralis minor, serratus anterior, and subclavius. Disruption can lead to referred pain and sensory disturbances in the anterior chest and upper extremities, as demonstrated in a correlation study involving 438 cervicogenic angina patients [18]. The chiropractic intervention, in this case, likely addressed the underlying cervical radiculopathy by relieving pressure on the affected nerve roots through spinal manipulation, soft tissue mobilization, and other manual therapies [7,8].

The second proposed mechanism for cervicogenic angina and dyspnea involves changes in sensorimotor integration in the prefrontal cortex. Cervicogenic pathology can also affect the prefrontal cortex by altering sensorimotor integration, wherein signals from the cervical spine can modulate prefrontal cortex activation [20], leading to perceived dyspnea and angina [21,22]. The prefrontal cortex helps process complex sensory information, modulating the respiratory and cardiac responses to perceived breathlessness and vasomotor cardiac drive [21,22]. Spinal manipulation, a common intervention in chiropractic care, has been shown to have neurophysiological effects, including altering neural input to the prefrontal cortex [20,23]. By alleviating cervical radiculopathy and restoring sensorimotor input, spinal manipulation may restore normal prefrontal cortex function and mitigate the dyspnea associated with cervicogenic pathology. Further research is needed to fully understand the mechanisms underlying the relationship between cervicogenic pathology, prefrontal cortex activation, compression of the cervical nerve roots, angina, and dyspnea, as well as the role of spinal manipulation in addressing these complex interactions.

Cervicogenic angina is not widely acknowledged and tends to be ignored in standard clinical procedures [12]. To ensure appropriate management and avoid unnecessary cardiac interventions, it is essential to distinguish between cervicogenic and cardiac symptoms [13]. To confirm the cervicogenic origin of the patient's symptoms, a comprehensive diagnostic evaluation should be performed, including the patient’s history, physical examination, and imaging studies [1]. This emphasizes the significance of thorough evaluation for accurate diagnosis and appropriate treatment. Conservative management, including chiropractic care, should be considered in patients presenting with cervicogenic angina and dyspnea, especially when imaging studies and clinical examinations suggest cervical spine involvement [12].

## Conclusions

In conclusion, this case study reports the successful chiropractic care of a patient with cervicogenic angina and dyspnea secondary to cervical radiculopathy. The patient experienced significant relief from angina-like symptoms and dyspnea after conservative treatment, emphasizing the importance of considering cervical spine involvement in patients presenting with chest pain and breathing difficulties. Accurate diagnosis and differentiation between cervicogenic and cardiac-related symptoms are crucial to ensure appropriate management and prevent unnecessary cardiac interventions. This case highlights the potential benefits of conservative management in patients with cervicogenic angina and dyspnea, further supporting the use of chiropractic care in the treatment of symptoms associated with cervical spine disorders. Further research is warranted to better understand the pathophysiology of cervicogenic angina and dyspnea, establish standardized diagnostic criteria, and develop evidence-based treatment protocols.
